# Prognostic value of the primary lesion apparent diffusion coefficient (ADC) in nasopharyngeal carcinoma: a retrospective study of 541 cases

**DOI:** 10.1038/srep12242

**Published:** 2015-07-17

**Authors:** Yuan Zhang, Xu Liu, Yun Zhang, Wen-Fei Li, Lei Chen, Yan-Ping Mao, Jing-Xian Shen, Fan Zhang, Hao Peng, Qing Liu, Ying Sun, Jun Ma

**Affiliations:** 1 Department of Radiation Oncology, Sun Yat-sen University Cancer Center, State Key Laboratory of Oncology in South China, Collaborative Innovation Center for Cancer Medicine, Canton, Guangdong Province, People’s Republic of China; 2 Imaging Diagnosis and Interventional Center, Sun Yat-sen University Cancer Center, State Key Laboratory of Oncology in South China, Collaborative Innovation Center for Cancer Medicine, Canton, Guangdong Province, People’s Republic of China; 3 Department of Medical Statistics and Epidemiology, School of Public Health, Sun Yat-sen University, Canton, People’s Republic of China

## Abstract

The prognostic value of the primary lesion pretreatment apparent diffusion coefficient (ADC), which is obtained by diffusion-weighted magnetic resonance imaging (MR-DWI), remains unknown in nasopharyngeal carcinoma (NPC). Thus, to investigate whether the pretreatment ADC value as measured from the primary site on MR-DWI is an independent prognostic factor in NPC, we retrospectively reviewed a cohort of 541 patients with histologically-proven stage I-IVB NPC. All patients underwent MRI using a 3-Tesla system (Trio Tim; Siemens, Erlangen Germany). To calculate ADC, the primary lesion was designated on the ADC map at the level of the largest tumor diameter to cover most of the lesion, avoiding cystic or necrotic components. Median and mean (±SD) pretreatment ADC were 0.713 and 0.716 ± 0.079 × 10^−3^ mm^2^/s, respectively. Univariate and multivariate analysis confirmed high pretreatment ADC was a good prognostic factor for poor local relapse-free survival and disease-free survival. Furthermore, the area under the ROC curve for prediction of local failure significantly increased when pretreatment ADC was combined with T classification (*P* = 0.004). Thus, pretreatment ADC might provide useful information for predicting outcome and selecting high-risk patients appropriate for more aggressive therapy. Further studies are warranted to investigate the biological basis of this observation.

According to the International Agency for Research on Cancer, there were over 86,000 new cases of nasopharyngeal cancer (NPC) worldwide in 2012; 80% of which occurred in Asia and only 6% in Europe^1^. NPC differs from other head and neck cancers because of its distinctly skewed geographic and ethnic distribution, its aggressive natural behavior, and specific therapeutic considerations. Unlike most other head and neck cancers, which are mainly treated by surgery, radiotherapy is the mainstay treatment modality for NPC^2^. Although local control has greatly improved in recent years with the application of magnetic resonance imaging (MRI) and intensity-modulated radiotherapy (IMRT), the management of recurrent NPC remains a challenging clinical problem due to high rates of fatal complications and poor survival as well as a low quality of life after retreatment^3^. For instance, it has been reported that up to 50% of patients with recurrent disease die of treatment-induced injuries after re-irradiation^4^. Therefore, it is of vital importance to be able to identify patients at high risk of local failure in order that individualized treatment can be offered.

“In-field” recurrence (95% of the recurrent tumor volume within the 95% isodose distribution of the radical dose) is the major local recurrence pattern in NPC^5^. One possible explanation is that tumors that recur possess different biological characteristics to tumors that do not^5^. Although the TNM staging system has been the cornerstone of the assessment of prognosis in NPC, it is merely based on the anatomic extent of the tumor^6^ and lacks an assessment of biological information. Thus, the exploration of parameters that reflect the intrinsic biological features of the tumor is highly important, and the prognostic value of these parameters demands urgent investigation.

Apparent diffusion coefficient (ADC) is a parameter obtained by diffusion-weighted magnetic resonance imaging (MR-DWI), a component of functional MRI, and reflects the Brownian movement of water molecules. ADC indirectly reflects the microvascular circulation, cell density and membrane integrity of the tissue^7^. A high pretreatment ADC value has been shown to be associated with a poor treatment response and/or survival after chemotherapy or chemoradiotherapy in many malignancies, including breast cancer^8^, hepatic metastases in colorectal cancer^9^ and some other head and neck cancers^10^,^11^,^12^,^13^. However, the prognostic value of the pretreatment ADC in NPC is unknown. Thus, we retrospectively analyzed a cohort of patients to investigate whether the pretreatment ADC value as measured from the primary lesion on diffusion weighted MRI is an independent prognostic factor in NPC.

## Patients and Methods

### Patients

This retrospective study was approved by the institutional Committee for Clinical Studies and the requirement for informed consent was waived. Between November 2010 and May 2012, 640 consecutive patients with histologically-proven stage I-IVB NPC were enrolled. Of these, 99 (15.5%) patients were subsequently excluded, including 8 (1.3%) cases due to a lack of clinical information, 28 (4.4%) because of poor imaging quality and 63 (10.0%) whose lesion was too small to be distinguished from normal tissues on DW images (tumor volume range, 0.68-6.36 ml). The remaining 541 patients were included (tumor volume range, 6.65–43.2 ml). The mean age of the entire cohort was 45.3 years (range, 14–74 years), with a male-to-female ratio of 2.6:1. The majority (99.6%) of patients were diagnosed with non-keratinizing carcinoma, while only 0.4% (2/541) were diagnosed with keratinizing squamous cell carcinoma.

All patients underwent a pretreatment evaluation including a complete patient history, physical examination, hematology and biochemistry profiles, MRI of the neck and nasopharynx, chest radiography, abdominal sonography and a whole body bone scan using single photon emission computed tomography. Furthermore, positron emission tomography-computed tomography (PET-CT) was performed on 156/541 (28.8%) patients. Medical records and imaging studies were analyzed retrospectively, and all patients were staged according to the 7th edition of the International Union against Cancer/American Joint Committee on Cancer (UICC/AJCC) system^6^. The characteristics of the patients analyzed in this study are shown in Table 1.

### Imaging

All patients underwent MRI using a 3-Tesla system (Trio Tim; Siemens, Erlangen Germany). The region from the suprasellar cistern to the inferior margin at the sternal end of the clavicle was examined with a head-and-neck coil. T1-weighted fast spin-echo images in the axial, coronal and sagittal planes (repetition time [TR]/echo time [TE], 650 ms/9 ms), T2-weighted fast spin-echo MR images in the axial plane (TR/TE, 2470 ms/90 ms) and a spin-echo echo-planar DWI sequence (matrix, 192 × 192; TR/TE, 5100 ms/96 ms; fov, 240; *b*-values, 0 and 1000 s/mm^2^; three signal averages) were obtained before contrast injection. After the intravenous administration of gadopentetate dimeglumine (0.1 mmol per kilogram of body weight; Magnevist, Schering, Berlin, Germany), axial and sagittal T1-weighted spin-echo sequences and coronal T1-weighted fat-suppressed spin-echo sequences were performed sequentially using the same parameters as applied before the injection of gadopentetate dimeglumine. Then, 5-mm thick sections were obtained with a 1-mm interslice gap for the axial plane, and 6-mm sections with a 1-mm interslice gap were obtained for the coronal and sagittal planes, resulting in a matrix size of 512 × 512.

**Figure 1:**
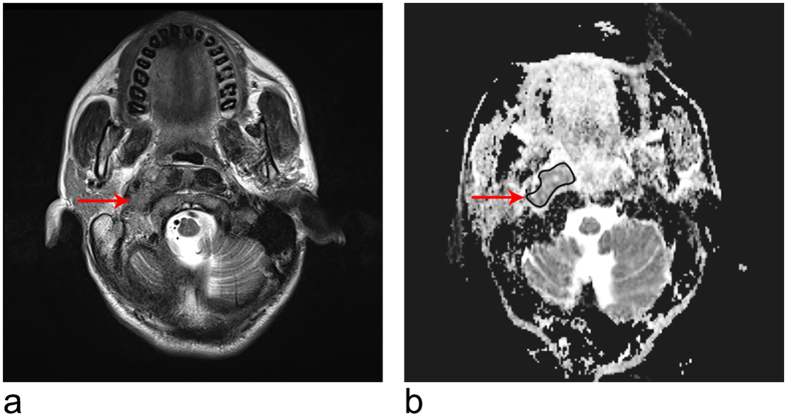
MR images of a 46-year-old woman. **(a)** Axial T2-weighted fast spin-echo (2470/90) MR image showing the primary tumor (arrow) in the nasopharynx with parapharyngeal extension. The spin-echo echo-planar (5100/96) DW images were obtained with *b* values of 0 and 1000 s/mm^2^. **(b)** The lesion (arrow) shows intermediate signal intensity on the corresponding ADC map (ADC = 0.893 × 10^−3^ mm^2^/s). The patient experienced local failure 22.8 months after treatment.

The ADC value was calculated as follows: the signal intensity (SI) of each pixel was fitted to the equation *SI = SI*_*0*_*e*^*-bD*^, where *SI* is the measured signal intensity, *b* is the *b-*value, *D* is the ADC, and *SI*_*0*_ is the *SI* at a *b*-value of 0. In this study, the ADC values were calculated using a *b*-value of 1,000 s/mm^2^. To calculate ADC, the primary lesion was designated as the region of interest (ROI) on the ADC map at the level of the largest tumor diameter to cover most of the lesion, avoiding cystic or necrotic components (with reference to T2-weighted and gadolinium-enhanced images). This procedure was carried out by two radiologists with more than 10 years of experience in MRI of head and neck cancer in consensus while blinded to the clinical outcomes. Representative ADC maps are shown in Fig. 1.

### Treatment

All patients were treated with IMRT; details of the techniques used at our center have previously been reported^14^. In total, 94.2% (377/400) of patients with stage III-IV A/B NPC received concurrent chemoradiotherapy ± neoadjuvant/adjuvant chemotherapy in conjunction with a platinum-based therapeutic clinical trial. When possible, salvage treatments such as brachytherapy, neck dissection and chemotherapy were provided in the event of documented relapse or persistent disease.

Patients were examined at least every 3 months during the first 2 years, and thereafter, a follow-up examination was performed every 5 months during years 3–5 or until death. Any residual disease found in the nasopharynx or cervical nodes within 6 months after completion of RT was regarded as local failure or regional failure, respectively. Distant failure was defined as the presence of metastases in locations beyond the regional nodes to which the cancer spread by vascular or lymphatic channels, such as the liver, bones or mediastinal lymph nodes^15^. Evaluations during follow-up included a complete patient history, physical examination, hematology and biochemistry profiles, MRI of the neck and nasopharynx, chest radiography, abdominal sonography and a whole body bone scan. All local recurrences were diagnosed by fibreoptic endoscopy and biopsy or MRI (or both) of the nasopharynx and the skull base showing progressive bone erosion and soft tissue swelling. Regional recurrences were diagnosed by clinical examination of the neck and, in doubtful cases, by fine needle aspiration or an MRI scan of the neck. Distant metastases were diagnosed by clinical symptoms, physical examinations, and imaging methods that included chest radiography, bone scan, MRI, CT and abdominal sonography.

### Statistical analysis

Statistical analyses were performed using SPSS version 22.0 (IBM Corporation, Armonk, NY, USA). The independent samples *t*-test was used to examine the differences in continuous variables between groups. The primary outcome of interest was local relapse-free survival (LRFS). Secondary outcomes included distant metastasis-free survival (DMFS), overall survival (OS) and disease-free survival (DFS). LRFS, DMFS and OS were calculated from the first day of treatment to the first local relapse, distant metastasis or death, respectively. DFS was defined as the latency to the date of disease progression or death from any cause. Actuarial rates were calculated using the Kaplan–Meier method and compared using the log-rank test. Multivariate analysis using a Cox proportional hazards model was used to test the independent significance of different factors by backward elimination. When testing the association with local failure, host factors (age and gender), tumor factors (T and N classification) and treatment method (radiotherapy alone or chemoradiotherapy) were included as variables in all analyses. Area under the receiver-operating characteristic (ROC) curves were used to select the optimal cut-off point for ADC by maximizing the conditional Youden score and to assess the prognostic validity of adding pretreatment ADC to the current T classification, based on the method described by Hanley^16^ and Zweig^17^. The criterion for statistical significance was set at α = 0.05 and all *P*-values were based on two-sided tests.

## Results

### Pretreatment ADC level and tumor staging

Of the entire cohort of 541 patients with NPC, the median and mean ± SD pretreatment ADC levels were 0.713 and 0.716 ± 0.79 × 10^−3^ mm^2^/s, respectively, (range, 0.498 to 0.958 × 10^−3^  mm^2^/s). The pretreatment ADC levels of patients with T3/4 disease (mean, 0.722 ± 0.080 × 10^−3^ mm^2^/s; median, 0.716 × 10^−3^ mm^2^/s; interquartile range, 0.672–0.776 × 10^−3^  mm^2^/s) were significantly higher than those of patients with T1/2 disease (mean, 0.704 ± 0.075 × 10^−3^ mm^2^/s; median, 0.702 × 10^−3^ mm^2^/s; interquartile range, 0.654–0.752 × 10^−3^ mm^2^/s; *P* = 0.014).

### Patterns of treatment failure

The median duration of follow-up for the entire cohort was 37.3 months (range, 4.5–52.0 months). A total of 23/541 (4.3%), 19/541 (3.5%), and 44/541 (7.8%) patients developed local failure, regional failure, and distant metastases, respectively; 25/541 (4.3%) patients died and 5/541 (0.7%) patients experienced both local-regional relapse and distant metastases. The 3-year LRFS, DMFS, OS and DFS rates were 95.7%, 92.5%, 95.3% and 86.0%, respectively.

### Prognostic value of pretreatment ADC in NPC

**Figure 2:**
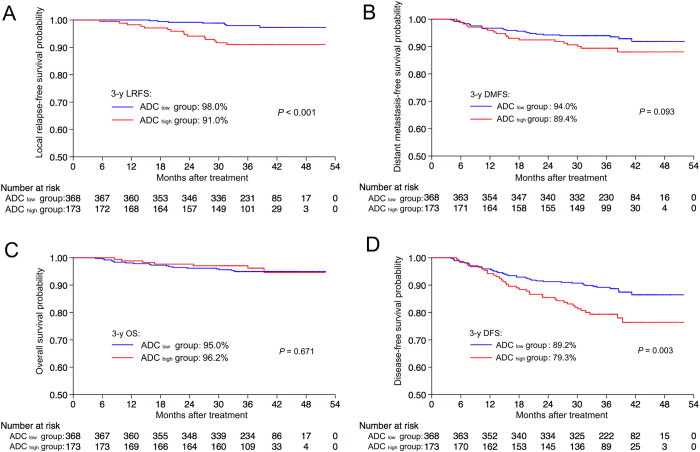
Kaplan-Meier curves of local relapse-free survival **(A)**, distant metastasis-free survival **(B)**, overall survival (**C**) and disease-free survival **(D)** for patients with NPC stratified as the ADC _low_ and ADC _high_ group. ADC _low_ group = patients with a primary lesion pretreatment ADC value < 0.747 × 10^−3^ mm^2^/s; ADC _high_ group = patients with a primary lesion pretreatment ADC value ≥ 0.747 × 10^−3^ mm^2^/s. Abbreviations: 3-y = 3-year; ADC = apparent diffusion coefficient; DFS = disease-free survival; DMFS = distant metastasis-free survival; LRFS = local relapse-free survival; OS = overall survival.

The optimal cut-off ADC value for local failure was 0.747 × 10^−3^  mm^2^/s (maximal conditional Youden score = 1.347; sensitivity, 65.2%; specificity, 69.5%; AUC [area under the ROC] = 0.68, *P = *0.004). This value was selected to classify patients into ADC _high_ (>0.747 × 10^−3^ mm^2^/s) and ADC _low_ (<0.747 × 10^−3^ mm^2^/s) groups. The Kaplan-Meier survival curves for the two groups are shown in Fig. 2. The 3-year LRFS (91.0% vs. 98.0%, *P* < 0.001; Fig. 2A) and DFS (79.3% vs. 89.2%, *P* = 0.003; Fig. 2D) rates for the ADC _high_ group were significantly lower than the corresponding rates for the ADC _low_ group. There were no differences in the 3-year DMFS (89.4% vs. 94.0%, *P* = 0.093; Fig. 2B) and OS (95.0% vs. 96.2%, P = 0.671; Fig. 2C) rates between groups.

Multivariate analysis was performed to adjust for confounding factors (seen in Table 2). Pretreatment ADC was found to be an independent prognostic factor for LRFS; advanced T classification (T3-4 vs. T1-2) was also associated with an increased risk of local failure, though this effect did not reach statistical significance. Meanwhile, pretreatment ADC and N classification were independent prognostic factors for DFS.

In patients with T3-4 disease, a high pretreatment ADC was also found to be a good prognostic factor for poor 3-year LRFS (89.0% vs. 97.2%, *P* = 0.003) and DFS (78.7% vs. 88.1%, *P* = 0.013) in univariate analysis. Furthermore, multivariate analysis confirmed that pretreatment ADC was an independent prognostic factor for LRFS and DFS.

### Prognostic validity of adding the pretreatment ADC of the primary lesion to the current T classification for local failure in NPC

**Figure 3:**
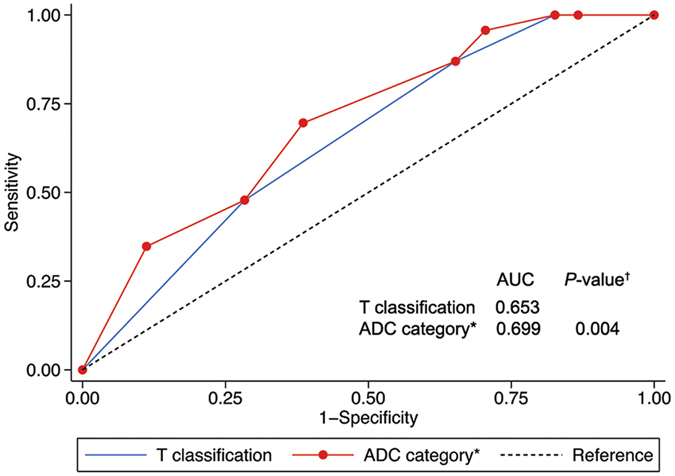
Receiver-operating characteristic curves for prediction of local failure after IMRT for the current T classification alone and the combination of the primary lesion pretreatment ADC value and T classification in patients with nasopharyngeal carcinoma (*n* = 541). *P*-values are compared to T classification alone. *Pretreatment ADC of the primary lesion (ADC ≥ 0.747 vs. ADC < 0.747 × 10^−3^ mm^2^/s) combined with T classification. Abbreviations: ADC = apparent diffusion coefficient; AUC = area under the curve.

Receiver-operating characteristic curves were used to evaluate the prognostic validity of adding the pretreatment ADC of the primary lesion to the T classification of the current TNM staging system for local failure in NPC. The AUC significantly increased when the pretreatment ADC (ADC ≥ 0.747 vs. ADC < 0.747 × 10^−3^ mm^2^/s) was added to T classification (0.699 vs. 0.653, *P* = 0.004; Fig. 3).

## Discussion

To the best of our knowledge, this is the first study to determine the prognostic value of the pretreatment ADC of the primary lesion in NPC. A high pretreatment ADC was associated with advanced T classification, the pretreatment ADC of the primary lesion was an independent prognostic indicator of LRFS and DFS, and combining the pretreatment ADC with the current T classification resulted in superior prognostic value for local failure compared to T classification alone in patients with NPC.

The mechanism underlying the association between a high pretreatment ADC value and local failure may be correlated with the radiosensitivity of the tumor. A number of biological features, such as hypoxia, inflammation, cell density and cell membrane integrity, could affect the diffusion of water in tissues and hence the ADC value^10^; these factors may also influence the radiosensitivity of the tumor. For example, hypoxia in the tumor promotes inflammation^18^, which is correlated with a high ADC value as it increases the interstitial water content of the tissue^19^. Moreover, hypoxia is a well-known characteristic that is associated with low levels of radiosensitivity^20^,^21^. Accordingly, tumors with low oxygen tensions may exhibit high ADC values and have a low level of radiosensitivity. On the other hand, ADC values are inversely correlated with cell density in many malignancies^22^,^23^,^24^, and tumors with a high cell density are more likely to have a higher number of viable proliferative cells and may exhibit a better response to radiotherapy^25^. Therefore, it is expected that tumors with fewer viable proliferative cells would have high ADC values and lower radiosensitivity. However, these mechanisms need to be confirmed by future studies.

Currently, TNM staging is the cornerstone of the assessment of prognosis and the establishment of treatment strategies in NPC, and the T classification is supposed to reflect the risk of local failure^26^. However, the TNM staging system is only based on the anatomical extent of the tumor^6^ and lacks biological information. Additionally, recent reports have suggested that the prognostic validity of the T classifications may have become blurred in the IMRT era^5^,^27^. The pretreatment ADC of the primary lesion may reflect the intrinsic biological characteristics of the primary tumor and provide additional useful information regarding T classification. This study demonstrated that the pretreatment ADC was an independent prognostic indicator of local failure and combination of the pretreatment ADC with T classification had superior prognostic value for local failure compared to T classification alone, suggesting that the pretreatment ADC can enhance the prognostic ability of the current T classification system. Patients with a high pretreatment ADC value might benefit from more aggressive treatment strategies, such as the addition of molecular-targeted agents or radiosensitizer agents such as sodium glycididazole^28^,^29^,^30^. However, the value of these strategies needs to be addressed in future studies.

MR-DWI enables non-invasive *in vivo* assessment of the intrinsic biological characteristics of tissues, and has proven value for differential diagnosis^31^, staging^32^, monitoring the response to therapy^7^,^33^, prognostic evaluation^10^,^34^ and early detection of recurrence^35^ in many malignancies. Several studies have demonstrated high pretreatment ADC values correlate with unfavorable treatment responses or poor survival after radiotherapy and/or chemotherapy in various tumor types including breast cancer^36^, brain tumor^37^, bladder cancer^38^ and some head and neck cancers^10^. Interestingly, one study in renal cancer reported that patients with low ADC values had a high risk of metastasis after surgery^39^; it remains unclear whether this effect was due to intrinsic tumor characteristics or the treatment modalities and this issue awaits further research. Moreover, large changes in the ADC value after treatment could be a useful indicator of treatment efficacy at an early phase, prior to the shrinkage of tumor volume, such as in breast cancer^40^ and brain tumors^41^, which provides compelling evidence of the potential of ADC in treatment decision making.

Similarly, in NPC, Chen *et al.* found that a large increase in ADC was associated with good treatment response after the first cycle of neoadjuvant chemotherapy in a cohort study of 31 patients with stage III-IV disease, while there was no significant difference in pretreatment ADC between responders and non-responders^42^. This was possibly due to the limited sample size. Similar results were observed in another study by the same group: Hong *et al.* did not find significant differences in pretreatment ADC between patients with and without residual tumors detected by MRI or biopsy three months after radiotherapy^7^. However, in Hong’s study, the proportion of residual tumors diagnosed by imaging only or histology was not clear, and all of the “residual tumors” detected by imaging may not actually contain viable tumor cells as the residual rate (16%) was much higher than the histological residual rate at the same time point in previous study (6.8% in patients treated with 2D-CRT)^43^. Besides, though Razek *et al.* reported that a low pretreatment ADC correlated with a large tumor volume and lymph node metastasis^44^, all patients enrolled were from non-endemic regions and only 53.3% (16/30) had undifferentiated non-keratinizing carcinoma (which accounted for up to 94.8% of cases in our study). Furthermore, the relationship between ADC changes and clinical outcome was not explored. Thus the correlation between pretreatment ADC and long-term survival outcomes in NPC was unknown. Here, we firstly analyzed the correlation of pretreatment ADC and clinical outcome in a large cohort of patients with NPC from the endemic region, and found out that pretreatment ADC was an independent prognostic factor for local control and disease-free survival, which is in accordance with findings in breast cancer^36^, some head and neck cancers^10^ and other tumor types^37^,^38^.

Nevertheless, the application of pretreatment ADC values in the clinic may be subject to a number of limitations. Firstly, detection of small primary lesions on DWI is usually difficult, although patients with small primary tumors have a low risk of local failure^27^. Secondly, these results may not be easily generalized to other centers as a result of inter-institutional differences in DWI techniques, including the *b*-values used. Moreover, no standard method has been established to determine the ADC value. Other methods described in the literature have included drawing multiple small regions of interest on one or several sections, or calculating ADC from the entire lesion. As MRI has been accepted as the imaging modality of choice for patients with NPC^45^, the standardization of DWI techniques and calculation methods could allow DWI to be more widely used in clinical settings. Thirdly, this study only included patients from the endemic region in China, of whom only 0.4% had keratinizing squamous cell carcinoma, which accounts for up to 67% of cases of NPC in western countries^46^. Finally, this study has unavoidable biases due to its retrospective nature. Prospective multi-center studies are required to confirm the results of this analysis.

In conclusion, the pretreatment ADC of the primary lesion was an independent prognostic indicator of local failure in patients with NPC, thus pretreatment ADC may provide useful information for predicting outcome and selecting high-risk patients appropriate for more aggressive therapy. Further studies are warranted to investigate the biological foundation of this observation.

## Additional Information

**How to cite this article**: Zhang, Y. *et al.* Prognostic value of the primary lesion apparent diffusion coefficient (ADC) in nasopharyngeal carcinoma: a retrospective study of 541 cases. *Sci. Rep.* **5**, 12242; doi: 10.1038/srep12242 (2015).

## Figures and Tables

**Table 1:** Clinical features of the 541 patients with NPC.

**Characteristic**	**No. of patients (%)**
**Age (years)**	
Median	45
Range	14–74
**Gender**	
Male	392 (72.4)
Female	149 (27.6)
**Pathologic type**^a^	
keratinizing squamous cell carcinoma	2 (0.4)
differentiated non-keratinizing carcinoma	26 (4.8)
undifferentiated non-keratinizing carcinoma	513 (94.8)
**T category**^b^	
T1	90 (16.6)
T2	93 (17.2)
T3	200 (36.9)
T4	158 (29.2)
**N category**^b^	
N0	83 (15.3)
N1	326 (60.3)
N2	87 (16.1)
N3	45 (8.0)
**Stage**^b^	
I	24 (4.4)
II	117 (21.6)
III	206 (38.1)
IV A/B	194 (35.9)
**Chemotherapy**	
Radiotherapy alone	67 (12.4)
Chemoradiotherapy	474 (87.6)

Abbreviations: NPC = nasopharyngeal carcinoma.

^a^Pathologic type: according to the 2005 World Health Organization classification of tumors^47^.

^b^According to the 7^th^ UICC/AJCC staging system^6^.

**Table 2:** Univariate and multivariate analyses of prognostic factors in the 541 patients with NPC.

Endpoint	Variable	Univariate analysis	Multivariate analysis	
		*P*-value	HR (95% CI)	*P*-value^a^
Local relapse-free survival	ADC	<0.001	3.858 (1.634–9.111)	0.002
	T classification^b^	0.031	3.153 (0.935–10.632)	0.064
Distant metastasis-free survival	N classification^b^	<0.001	3.139 (1.736–5.675)	<0.001
Disease-free survival	ADC	0.003	1.829 (1.177–2.843)	0.007
	N classification^b^	<0.001	2.386 (1.526–3.731)	<0.001

Abbreviations: ADC = apparent diffusion coefficient; CI = confidence interval; HR = hazard ratio; NPC = nasopharyngeal carcinoma.

^a^*P*-values were calculated using an adjusted Cox proportional hazards model.

^b^According to the 7^th^ UICC/AJCC staging system^6^.
